# 7-Hy­droxy-8-isopropyl-1,1,4a-trimethyl-4a,9,10,10a-tetra­hydro-phenanthren-2(1*H*)-one

**DOI:** 10.1107/S1600536811009561

**Published:** 2011-03-19

**Authors:** Ahmed Benharref, Essêdiya Lassaba, Noureddine Mazoir, Jean-Claude Daran, Moha Berraho

**Affiliations:** a Laboratoire de Chimie Biomoléculaires, Substances Naturelles et Réactivité, URAC16, Université Cadi Ayyad, Faculté des Sciences Semlalia, BP 2390 Bd My Abdellah, 40000 Marrakech, Morocco; b Laboratoire de Chimie de Coordination, 205 route de Narbonne, 31077 Toulouse Cedex 04, France

## Abstract

The title compound, C_20_H_26_O_2_, was isolated from a chloro­form extract of *Tetra­clinis articulata* wood. The mol­ecule contains three fused rings which exhibit different conformations. The non-aromatic oxo-substituted ring has a screw-boat conformation, while the central ring has a half-chair conformation. In the crystal, mol­ecules are linked to each other by inter­molecular O—H⋯O hydrogen bonds involving the carbonyl and hy­droxy groups.

## Related literature

For background to the biological activity of diterpenoids, see: Atta-ur-Rahman & Choudhary (1999 ▶); Azucena & Mobashery (2001 ▶); Panter *et al.* (2002 ▶); Ulusu *et al.* (2002 ▶). For their use in traditional medicine, see: Bellakhdar (1997 ▶) and for their medicinal properties, see: Barrero *et al.* (2003 ▶); Comte *et al.* (1995 ▶); Evidente *et al.* (1997 ▶). For the synthesis see: Zeroual *et al.* (2007 ▶). For conformational analysis, see: Cremer & Pople (1975 ▶).
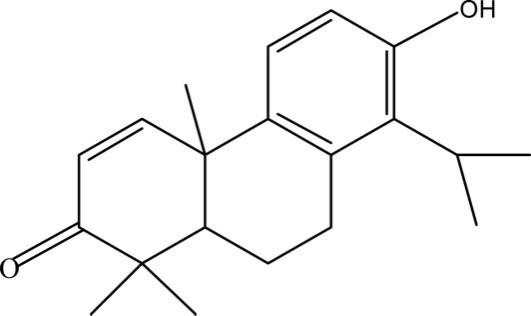


## Experimental

### 

#### Crystal data

C_20_H_26_O_2_
*M* *_r_* = 298.41
Monoclinic, 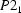

*a* = 11.6731 (8) Å
*b* = 6.4314 (4) Å
*c* = 12.1488 (10) Å
β = 111.592 (9)°
*V* = 848.06 (11) Å^3^
*Z* = 2
Mo *K*α radiation
μ = 0.07 mm^−1^
*T* = 180 K
0.48 × 0.36 × 0.29 mm

#### Data collection

Agilent Xcalibur Eos Gemini ultra diffractometer
9252 measured reflections
1893 independent reflections
1591 reflections with *I* > 2σ(*I*)
*R*_int_ = 0.066

#### Refinement

*R*[*F* ^2^ > 2σ(*F* ^2^)] = 0.047
*wR*(*F* ^2^) = 0.128
*S* = 1.03
1893 reflections
207 parameters
1 restraint
H-atom parameters constrained
Δρ_max_ = 0.18 e Å^−3^
Δρ_min_ = −0.22 e Å^−3^

### 

Data collection: *CrysAlis PRO* (Agilent, 2010 ▶); cell refinement: *CrysAlis PRO*; data reduction: *CrysAlis PRO*; program(s) used to solve structure: *SHELXS97* (Sheldrick, 2008 ▶); program(s) used to refine structure: *SHELXL97* (Sheldrick, 2008 ▶); molecular graphics: *ORTEP-3 for Windows* (Farrugia, 1997 ▶) and *PLATON* (Spek, 2009 ▶); software used to prepare material for publication: *WinGX* (Farrugia, 1999 ▶).

## Supplementary Material

Crystal structure: contains datablocks global, I. DOI: 10.1107/S1600536811009561/fj2405sup1.cif

Structure factors: contains datablocks I. DOI: 10.1107/S1600536811009561/fj2405Isup2.hkl

Additional supplementary materials:  crystallographic information; 3D view; checkCIF report

## Figures and Tables

**Table 1:** Hydrogen-bond geometry (Å, °)

*D*—H⋯*A*	*D*—H	H⋯*A*	*D*⋯*A*	*D*—H⋯*A*
O2—H2⋯O1^i^	0.84	2.03	2.791 (3)	150

Symmetry code: (i) 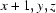.

**Fig. 1.**
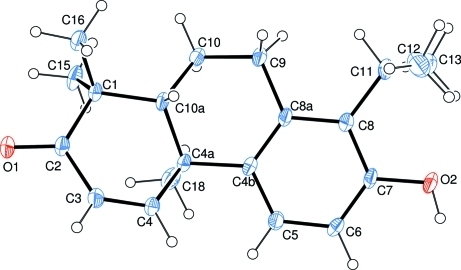
: Molecular structure of the title compound with the atom-labelling scheme. Displacement ellipsoids are drawn at the 30% probability. level. H atoms are represented as small spheres of arbitrary radii.

**Fig. 2.**
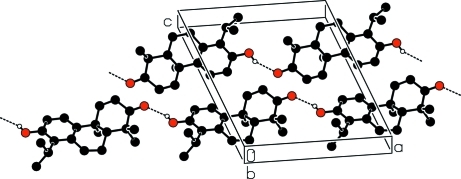
: Partial packing view showing the O—H···O interactions (dashed lines) and the formation of a chain parallel to the b axis. H atoms not involved in hydrogen bonding have been omitted for clarity. [Symmetry code:(i) x + 1, y, z]

## supplementary crystallographic information

### Comment

Among the diterpenoids, the class with the phenantrene skeleton may have biological activities which have been recently reported (Atta-ur-Rahman *et al.*,1999; Azucena *et al.*,2001; Panter *et al.*, 2002; Ulusu *et al.*, 2002). These diterpenoids are often isolated from the medicinal plants. In our study, we were interested to the medicinal plant, Tetraclinis articulata which is used in Moroccan traditional medicine (Bellakhdar, 1997). Some of its oxygenated compounds are effective as an antifongic (Evidente *et al.*, 1997), cytotoxic (Comte *et al.*, 1995) and inhibit various human leukocyte functions(Barrero *et al.*, 2003). In order to isolate similar compounds, we have studied the chloroform extract of Tetraclinis articulata wood. Thus, the extraction with chloroform using a soxhlet apparatus allow us isolate the totarolenone (7-hydroxy-8-isporpyl-1,1,4a-trimethyl- 4a,9,10,10*a*- tetrahydro-1*H*-phenanthren-3-one) a derivative of totarolone (7-hydroxy-8-isporpyl-1,1,4a-trimethyl-3,4,4a,9,10,10*a*- hexahydophenantren-2-one) (Zeroual *et al.*, 2007). The structure of this new product was determined by NMR spectral analysis of 1H, 13 C and mass spectroscopy and confirmed by its single-crystal X-ray structure. The molecule (I) is built up from three fused six-membered rings. The non aromatic oxo-substituted ring has a screw boat conformation, as indicated by the total puckering amplitude QT = 0.490 (3)Å and spherical polar angle θ =67.1 (4)° with φ = 272.2 (3)°. While the central ring has a half chair conformation with QT = 0.545 (3) Å, θ =53.4 (3)°, φ = 278.5 (3)° (Cremer & Pople, 1975). Molecules are linked by intermolecular O—H···O hydrogen bonds (Table 1, Figure 2) involving the carbonyl and the hydroxy groups and propagate in chain parallel to the *a* axis.

### Experimental

50 g of wood powder Tetraclinis articulata was extracted with chloroform in Soxhlet apparatus during 24 h. After evaporating the solvent under reduced pressure, a residue of 3.2 g was obtained. Chromatography on a column of silica gel of this residue eluting with hexan - ethyl acetate (94 / 4), allowed us to isolate in pure form 640 mg of 7-hydroxy-8-isporpyl- 1, 1,4 a-trimethyl-4a, 9,10,10 a-tetrahydro-1*H*-phenanthrene-3-one. Crystallization of this product was performed at room temperature in a solution of ethyl acetate.

### Refinement

All H atoms were fixed geometrically and treated as riding with C—H = 0.96 Å (methyl), 0.97 Å (methylene), 0.98Å (methine) with *U*_iso_(H) = 1.2U_eq_ (methylene, methine) or *U*_iso_(H) = 1.5*U*_eq_ (methyl). In the absence of significant anomalous scattering, the absolute configuration could not be reliably determined and thus 1554 Friedel pairs were merged and any references to the Flack parameter were removed.

### Crystal data

**Table:**

C_20_H_26_O_2_	*F*(000) = 324
*M**_r_* = 298.41	*D*_x_ = 1.169 Mg m^−^^3^
Monoclinic, *P*2_1_	Mo *K*α radiation, λ = 0.71073 Å
Hall symbol: P 2yb	Cell parameters from 3905 reflections
*a* = 11.6731 (8) Å	θ = 3.6–29.2°
*b* = 6.4314 (4) Å	µ = 0.07 mm^−^^1^
*c* = 12.1488 (10) Å	*T* = 180 K
β = 111.592 (9)°	Prism, colourless
*V* = 848.06 (11) Å^3^	0.48 × 0.36 × 0.29 mm
*Z* = 2	

### Data collection

**Table:**

Agilent Xcalibur Eos Gemini ultra diffractometer	1591 reflections with *I* > 2σ(*I*)
Radiation source: Enhance (Mo) X-ray Source	*R*_int_ = 0.066
graphite	θ_max_ = 26.4°, θ_min_ = 3.7°
Detector resolution: 16.1978 pixels mm^-1^	*h* = −14→14
ω scans	*k* = −8→8
9252 measured reflections	*l* = −15→15
1893 independent reflections	

### Refinement

**Table:**

Refinement on *F*^2^	Primary atom site location: structure-invariant direct methods
Least-squares matrix: full	Secondary atom site location: difference Fourier map
*R*[*F*^2^ > 2σ(*F*^2^)] = 0.047	Hydrogen site location: inferred from neighbouring sites
*wR*(*F*^2^) = 0.128	H-atom parameters constrained
*S* = 1.03	*w* = 1/[σ^2^(*F*_o_^2^) + (0.0659*P*)^2^ + 0.2266*P*] where *P* = (*F*_o_^2^ + 2*F*_c_^2^)/3
1893 reflections	(Δ/σ)_max_ < 0.001
207 parameters	Δρ_max_ = 0.18 e Å^−^^3^
1 restraint	Δρ_min_ = −0.22 e Å^−^^3^

### Special details

**Table:**

Geometry. All s.u.'s (except the s.u. in the dihedral angle between two l.s. planes) are estimated using the full covariance matrix. The cell s.u.'s are taken into account individually in the estimation of s.u.'s in distances, angles and torsion angles; correlations between s.u.'s in cell parameters are only used when they are defined by crystal symmetry. An approximate (isotropic) treatment of cell s.u.'s is used for estimating s.u.'s involving l.s. planes.
Refinement. Refinement of *F*^2^ against ALL reflections. The weighted *R*-factor *wR* and goodness of fit *S* are based on *F*^2^, conventional *R*-factors *R* are based on *F*, with *F* set to zero for negative *F*^2^. The threshold expression of *F*^2^ > σ(*F*^2^) is used only for calculating *R*-factors(gt) *etc*. and is not relevant to the choice of reflections for refinement. *R*-factors based on *F*^2^ are statistically about twice as large as those based on *F*, and *R*- factors based on ALL data will be even larger.

### Fractional atomic coordinates and isotropic or equivalent isotropic displacement parameters (Å^2^)

**Table:**

	*x*	*y*	*z*	*U*_iso_*/*U*_eq_	
C1	−0.3286 (2)	0.3531 (5)	0.7146 (2)	0.0290 (6)	
C2	−0.4104 (2)	0.3898 (5)	0.5849 (3)	0.0314 (7)	
C3	−0.3525 (3)	0.4063 (6)	0.4963 (3)	0.0364 (7)	
H3	−0.4016	0.3822	0.4154	0.045 (10)*	
C4	−0.2337 (3)	0.4540 (5)	0.5251 (3)	0.0324 (7)	
H4	−0.2019	0.4667	0.4638	0.047 (10)*	
C4A	−0.1473 (2)	0.4886 (5)	0.6526 (2)	0.0283 (6)	
C4B	−0.0133 (2)	0.4386 (4)	0.6696 (2)	0.0274 (6)	
C5	0.0337 (2)	0.5177 (5)	0.5876 (3)	0.0296 (6)	
H5	−0.0179	0.6000	0.5237	0.036*	
C6	0.1537 (2)	0.4788 (5)	0.5974 (3)	0.0303 (7)	
H6	0.1846	0.5371	0.5420	0.036*	
C7	0.2287 (2)	0.3544 (5)	0.6887 (2)	0.0299 (6)	
C8	0.1866 (2)	0.2729 (5)	0.7742 (2)	0.0287 (6)	
C8A	0.0651 (2)	0.3211 (5)	0.7647 (2)	0.0273 (6)	
C9	0.0224 (2)	0.2470 (6)	0.8626 (3)	0.0351 (7)	
H9A	0.0867	0.2814	0.9402	0.042*	
H9B	0.0136	0.0938	0.8579	0.042*	
C10	−0.0995 (2)	0.3422 (6)	0.8568 (2)	0.0336 (7)	
H10A	−0.1329	0.2626	0.9079	0.040*	
H10B	−0.0861	0.4875	0.8856	0.040*	
C10A	−0.1905 (2)	0.3373 (5)	0.7292 (2)	0.0282 (6)	
H10	−0.1817	0.1953	0.6997	0.034*	
C11	0.2702 (3)	0.1345 (6)	0.8719 (3)	0.0383 (8)	
H11	0.2229	0.0978	0.9231	0.046*	
C12	0.3000 (3)	−0.0718 (6)	0.8246 (4)	0.0526 (9)	
H12A	0.3534	−0.0452	0.7800	0.079*	
H12B	0.2233	−0.1371	0.7725	0.079*	
H21A	0.3421	−0.1649	0.8911	0.079*	
C13	0.3870 (3)	0.2455 (7)	0.9521 (3)	0.0474 (9)	
H13A	0.4396	0.2761	0.9071	0.071*	
H13B	0.4317	0.1559	1.0195	0.071*	
H13C	0.3646	0.3757	0.9810	0.071*	
C15	−0.3538 (3)	0.5228 (6)	0.7922 (3)	0.0422 (8)	
H15A	−0.4422	0.5272	0.7773	0.063*	
H15B	−0.3274	0.6580	0.7727	0.063*	
H15C	−0.3079	0.4913	0.8758	0.063*	
C16	−0.3680 (3)	0.1419 (5)	0.7484 (3)	0.0371 (7)	
H16A	−0.3634	0.0355	0.6925	0.056*	
H16B	−0.4528	0.1515	0.7457	0.056*	
H16C	−0.3130	0.1042	0.8286	0.056*	
C18	−0.1553 (3)	0.7207 (5)	0.6809 (3)	0.0427 (8)	
H18A	−0.1174	0.8047	0.6362	0.064*	
H18B	−0.1117	0.7442	0.7658	0.064*	
H18C	−0.2419	0.7606	0.6587	0.064*	
O1	−0.52293 (18)	0.4045 (4)	0.5543 (2)	0.0440 (6)	
O2	0.34674 (17)	0.3068 (4)	0.69940 (18)	0.0396 (6)	
H2	0.3623	0.3635	0.6442	0.059*	

### Atomic displacement parameters (Å^2^)

**Table:**

	*U*^11^	*U*^22^	*U*^33^	*U*^12^	*U*^13^	*U*^23^
C1	0.0193 (12)	0.0352 (16)	0.0359 (15)	0.0034 (12)	0.0140 (11)	0.0023 (13)
C2	0.0210 (13)	0.0368 (17)	0.0389 (15)	0.0037 (12)	0.0138 (11)	0.0033 (13)
C3	0.0240 (14)	0.056 (2)	0.0299 (14)	0.0060 (14)	0.0106 (11)	0.0032 (14)
C4	0.0247 (14)	0.0416 (18)	0.0355 (15)	0.0089 (12)	0.0165 (12)	0.0062 (13)
C4A	0.0213 (13)	0.0343 (16)	0.0338 (14)	0.0029 (12)	0.0153 (11)	0.0026 (13)
C4B	0.0208 (13)	0.0313 (16)	0.0338 (14)	−0.0026 (11)	0.0145 (11)	−0.0017 (12)
C5	0.0245 (14)	0.0317 (15)	0.0345 (14)	0.0003 (12)	0.0129 (11)	0.0013 (12)
C6	0.0258 (14)	0.0365 (17)	0.0346 (14)	−0.0068 (13)	0.0182 (12)	−0.0015 (13)
C7	0.0166 (12)	0.0403 (17)	0.0354 (15)	−0.0036 (13)	0.0126 (11)	−0.0034 (14)
C8	0.0204 (13)	0.0335 (15)	0.0335 (14)	−0.0016 (12)	0.0115 (11)	0.0003 (12)
C8A	0.0180 (12)	0.0329 (15)	0.0334 (14)	−0.0017 (11)	0.0125 (11)	0.0000 (12)
C9	0.0195 (14)	0.0533 (19)	0.0340 (15)	−0.0013 (13)	0.0117 (11)	0.0041 (14)
C10	0.0235 (13)	0.0494 (19)	0.0310 (14)	−0.0024 (14)	0.0136 (11)	0.0025 (14)
C10A	0.0171 (12)	0.0396 (16)	0.0309 (14)	0.0030 (12)	0.0124 (10)	0.0005 (13)
C11	0.0193 (13)	0.055 (2)	0.0408 (16)	0.0001 (14)	0.0119 (12)	0.0067 (15)
C12	0.0414 (19)	0.049 (2)	0.061 (2)	0.0066 (16)	0.0101 (17)	0.0116 (18)
C13	0.0300 (17)	0.069 (3)	0.0414 (18)	−0.0075 (16)	0.0115 (14)	0.0022 (18)
C15	0.0279 (16)	0.053 (2)	0.054 (2)	−0.0006 (15)	0.0258 (15)	−0.0119 (17)
C16	0.0241 (14)	0.0423 (18)	0.0495 (18)	0.0017 (13)	0.0187 (13)	0.0097 (15)
C18	0.0426 (19)	0.0361 (19)	0.061 (2)	0.0025 (14)	0.0327 (17)	0.0003 (16)
O1	0.0197 (10)	0.0687 (17)	0.0459 (12)	0.0077 (10)	0.0146 (9)	0.0109 (12)
O2	0.0212 (10)	0.0626 (16)	0.0409 (12)	0.0021 (10)	0.0184 (9)	0.0044 (11)

### Geometric parameters (Å, °)

**Table:**

C1—C2	1.531 (4)	C9—H9A	0.9900
C1—C16	1.538 (4)	C9—H9B	0.9900
C1—C15	1.539 (4)	C10—C10A	1.523 (4)
C1—C10A	1.559 (3)	C10—H10A	0.9900
C2—O1	1.230 (3)	C10—H10B	0.9900
C2—C3	1.471 (4)	C10A—H10	1.0000
C3—C4	1.336 (4)	C11—C13	1.530 (5)
C3—H3	0.9500	C11—C12	1.536 (5)
C4—C4A	1.522 (4)	C11—H11	1.0000
C4—H4	0.9500	C12—H12A	0.9800
C4A—C4B	1.535 (4)	C12—H12B	0.9800
C4A—C18	1.542 (4)	C12—H21A	0.9800
C4A—C10A	1.554 (4)	C13—H13A	0.9800
C4B—C5	1.397 (4)	C13—H13B	0.9800
C4B—C8A	1.401 (4)	C13—H13C	0.9800
C5—C6	1.385 (4)	C15—H15A	0.9800
C5—H5	0.9500	C15—H15B	0.9800
C6—C7	1.386 (4)	C15—H15C	0.9800
C6—H6	0.9500	C16—H16A	0.9800
C7—O2	1.371 (3)	C16—H16B	0.9800
C7—C8	1.403 (4)	C16—H16C	0.9800
C8—C8A	1.414 (4)	C18—H18A	0.9800
C8—C11	1.517 (4)	C18—H18B	0.9800
C8A—C9	1.526 (4)	C18—H18C	0.9800
C9—C10	1.526 (4)	O2—H2	0.8400
			
C2—C1—C16	106.1 (2)	C9—C10—H10A	109.8
C2—C1—C15	109.5 (2)	C10A—C10—H10B	109.8
C16—C1—C15	108.7 (2)	C9—C10—H10B	109.8
C2—C1—C10A	110.7 (2)	H10A—C10—H10B	108.3
C16—C1—C10A	108.1 (2)	C10—C10A—C4A	109.7 (2)
C15—C1—C10A	113.5 (2)	C10—C10A—C1	114.8 (2)
O1—C2—C3	120.0 (3)	C4A—C10A—C1	116.2 (2)
O1—C2—C1	121.0 (2)	C10—C10A—H10	104.9
C3—C2—C1	119.0 (2)	C4A—C10A—H10	104.9
C4—C3—C2	122.5 (3)	C1—C10A—H10	104.9
C4—C3—H3	118.8	C8—C11—C13	113.0 (3)
C2—C3—H3	118.8	C8—C11—C12	112.5 (3)
C3—C4—C4A	122.4 (2)	C13—C11—C12	111.7 (3)
C3—C4—H4	118.8	C8—C11—H11	106.3
C4A—C4—H4	118.8	C13—C11—H11	106.3
C4—C4A—C4B	111.5 (2)	C12—C11—H11	106.3
C4—C4A—C18	107.3 (3)	C11—C12—H12A	109.5
C4B—C4A—C18	108.4 (3)	C11—C12—H12B	109.5
C4—C4A—C10A	106.2 (2)	H12A—C12—H12B	109.5
C4B—C4A—C10A	109.2 (2)	C11—C12—H21A	109.5
C18—C4A—C10A	114.4 (2)	H12A—C12—H21A	109.5
C5—C4B—C8A	118.4 (2)	H12B—C12—H21A	109.5
C5—C4B—C4A	118.4 (2)	C11—C13—H13A	109.5
C8A—C4B—C4A	123.2 (2)	C11—C13—H13B	109.5
C6—C5—C4B	121.5 (3)	H13A—C13—H13B	109.5
C6—C5—H5	119.2	C11—C13—H13C	109.5
C4B—C5—H5	119.2	H13A—C13—H13C	109.5
C5—C6—C7	119.6 (2)	H13B—C13—H13C	109.5
C5—C6—H6	120.2	C1—C15—H15A	109.5
C7—C6—H6	120.2	C1—C15—H15B	109.5
O2—C7—C6	121.4 (2)	H15A—C15—H15B	109.5
O2—C7—C8	117.4 (2)	C1—C15—H15C	109.5
C6—C7—C8	121.2 (2)	H15A—C15—H15C	109.5
C7—C8—C8A	118.1 (3)	H15B—C15—H15C	109.5
C7—C8—C11	119.9 (2)	C1—C16—H16A	109.5
C8A—C8—C11	122.0 (2)	C1—C16—H16B	109.5
C4B—C8A—C8	121.1 (2)	H16A—C16—H16B	109.5
C4B—C8A—C9	120.7 (2)	C1—C16—H16C	109.5
C8—C8A—C9	118.2 (2)	H16A—C16—H16C	109.5
C8A—C9—C10	113.9 (3)	H16B—C16—H16C	109.5
C8A—C9—H9A	108.8	C4A—C18—H18A	109.5
C10—C9—H9A	108.8	C4A—C18—H18B	109.5
C8A—C9—H9B	108.8	H18A—C18—H18B	109.5
C10—C9—H9B	108.8	C4A—C18—H18C	109.5
H9A—C9—H9B	107.7	H18A—C18—H18C	109.5
C10A—C10—C9	109.2 (2)	H18B—C18—H18C	109.5
C10A—C10—H10A	109.8	C7—O2—H2	109.5

### Hydrogen-bond geometry (Å, °)

**Table:**

*D*—H···*A*	*D*—H	H···*A*	*D*···*A*	*D*—H···*A*
O2—H2···O1^i^	0.84	2.03	2.791 (3)	150

Symmetry codes: (i) *x*+1, *y*, *z*.
